# Oral Health Status of Children with Autistic Spectrum Disorder Compared with Non-authentic Peers

**Published:** 2017-11

**Authors:** Işıl Özgül KALYONCU, Ilknur TANBOGA

**Affiliations:** Dept. of Dentistry, School of Pediatric Dentistry, Marmara University, Istanbul, Turkey

## Dear Editor-in-Chief

Autistic spectrum disorder (ASD) is characterized by impairments in communication and social relationships and a restricted, repetitive and stereotyped repertoire of activities, behaviors and interests. Therefore, affected children may be incapable of cooperating in the dental setting, reducing their access to dental care (1,2). This study was designed to explore the oral hygiene practices and oral health status of children with ASD and factors that may affect their oral health compared with non-autistic peers as a control group.

Sixty children with ASD (mean age=10.8±2.4, range: 6–14) and 60 unaffected primary school students (mean age=10.5±2.3, range: 6–14) from the Dentistry Department, School of Dentistry Paediatric, Marmara University, Istanbul, Turkey participated in the study with their parents. Children were orally examined by the dentist using disposable dental mirrors and wooden tongue spatulas in classrooms during broad daylight. The parents or caregivers were interviewed to complete a questionnaire designed to collect data about oral health practices. Dental caries lesions were registered according to the WHO criteria. Modified bacterial dental plaque and gingival health indices were used to assess periodontal health.

Informed consent was taken from the parents of subjects and the study was approved by the university.

In the study population, the mean DMF-T and df-t values of the children with ASD were 2±2.26 and 1.65±2.52, respectively. The mean DMF-T and df-t values of the non-autistic children were 2.22±1.78 and 2.27±2.75, respectively. Dental caries lesions were detected in 39 (65%) of the children with ASD and 54 (90%) of the non-autistic children, and this difference was significant (*P*=0.001). An intra-group comparison revealed a significant increase in the DMF-T score with increasing age in children with ASD (r=0.462, *P*=0.000). In all, 67% of parents in the ASD group reported that they use snacks for positive reinforcement while educating their children and no significant relation was found between the occurrence of caries and the use of snacks as positive reinforcement. The tooth brushing habits of the two groups are shown in Table 1.

**Table 1:** Tooth brushing characteristics of the study population

	***Tooth brushing***	***By himself/herself***			***By parents***	
		***n %***			***n %***	
		***1/day***	***2/day***	***3/day***	***1/day***	***2/day***
Childen with ASD	40 (67)	6 (10)	10 (17)	0.0	20 (33)	4 (7)
Non-autistic children	54 (90)	19 (33)	28 (47)	6 (10)	1 (2)	0.0

**Fig. 1:**
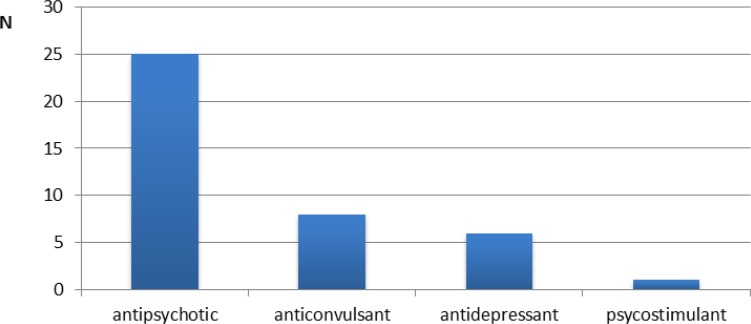
Medications prescribed for the long-term treatment of children with ASD

Significantly more non-autistic children brushed their teeth at least once a day (*P*=0.002); however, tooth brushing was performed by the parents of children with ASD significantly (*P*=0.000) more than by the parents of non-autistic children. The children with ASD had significantly higher bacterial dental plaque scores than children in the control group (*P*<0.045). The number of children with healthy gingivae was significantly (*P*<0.0242) greater in the control group than in the ASD group. Half the parents of children with ASD reported that their children are prescribed different types of medications (Fig. 1). The prevalence of caries was significantly higher in children with ASD prescribed medicine (*P*=0.002). While not significant, there was a tendency for children with ASD under long-term treatment with prescribed medication to have increased localized/generalized gingivitis (*P*=0.071). Caries susceptibility was lower in people with autism than in those without autism, while the caries prevalence was similar in both groups (2,3).

The prevalence of caries was lower in children with ASD than in their peers, despite the fact that snacks are used as rewards in the Special Personal Training Program, which is part of their training; this result may be explained by the families and trainers limiting and controlling the consumption of these foods, which have high potential for causing caries. Additionally, the good supervision of the parents regarding tooth brushing is an extremely large factor in children with ASD having fewer caries. The oral hygiene status of patients with autism was lower than that of unaffected patients (4). Patients with autism have a lower hygiene level but a comparable caries index compared with patients without autism (1,4). Poor oral hygiene and periodontal problems could be related to irregular brushing habits resulting from the difficulties encountered by trainers and parents while brushing these children’s teeth. Another possible explanation for the presence of generalized gingivitis could be related to the side effects of medications. As the children with ASD increasing by the age, the number of caries is increasing; this finding is related to both the increasing number of permanent teeth and tooth brushing by family members becoming increasingly difficult with increasing age.

The most commonly prescribed medications were antipsychotic drugs (2,5). We found that the number of children with caries were greater in the group of children with ASD treated with prescription medication. This finding could potentially be explained by the high potential carcinogenicity of sweeteners used in syrups, the side effects of other medications, or the drinks used to swallow tablets. It would be meaningful regarding the oral health of children with ASD to determine how many of their medications are sugar-based or taken with sweet drinks.

Although ASD was not associated with the prevalence or severity of increased dental caries, dental professionals need to emphasize the importance of regular preventive measures, such as using topical fluoride gels or daily rinses and good dietary habits.
